# The Treatment at Nordrach

**Published:** 1901-07-13

**Authors:** 


### THE TREATMENT AT NORDRACH.

In the *Practitioner* for this month Dr. R. Mander Smyth gives a striking account of his personal experience of "galloping consumption" and of the benefits he derived from treatment at Nordrach. That he was in a very desperate condition admits of no doubt; and it is clear that he nearly lost his life from an acute exacerbation of the tuberculous process set up by the over-exertion of the journey; yet as the result of the rest, the open air, and the hypernutrition he made a good "recovery." He says that the moral of his case is not far to seek. The open-air treatment at home had been a failure because the feeding was inadequate, the rest was not absolute—to be out of bed with a temperature indicating activity of the tuberculous process was madness—and, lastly, his room was insufficiently flushed with air. At Nordrach the isolation from sympathetic friends who made him talk he found an absolute relief. The solid diet and the absolute peace in the open air soon produced an effect on the temperature in spite of the fulminating effect of the journey. His friends were sent away, and he only saw the doctor and a nurse, who was enjoined never to let him talk. The windows constituting nearly the whole of one side of his room were open day and night, and a strong draught played through them. He was occasionally bombarded by summer hailstorms, and at times in the winter waked up to find that an inch of snow had silently drifted upon his bed. But far more important, as he thinks, than the fresh air were the meals. "Three a day—at eight, one, and seven—they came with pitiless regularity. A plate of pork and potatoes, a leg and wing of a chicken, salad and more potatoes, and to finish a large wedge of very solid pastry, would constitute a typical dinner; no more, indeed, than a man of hearty appetite would consume, but to the feeble invalid a Gargantuan repast." This during the time when he was in his room; but when at last he was sufficiently recovered to go to the dining-room he often spent two or three hours in eating his dinner. He pays a high tribute to his doctor, and we are led to feel more than ever how largely the good results of sanatorium treatment depend on the exact apportionment of the treatment to the varying exigencies of the cases.

